# The Role of Interaction Model in Simulation of Drug Interactions and QT Prolongation

**DOI:** 10.1007/s40495-016-0075-9

**Published:** 2016-10-27

**Authors:** Barbara Wiśniowska, Sebastian Polak

**Affiliations:** 1 Unit of Pharmacoepidemiology and Pharmacoeconomics, Faculty of Pharmacy, Medical College, Jagiellonian University, Medyczna 9 Street, 30-688 Kraków, Poland; 2 Simcyp (part of Certara), Blades Enterprise Centre John Street, Sheffield, S2 4SU UK

**Keywords:** PBPK models, Drug interactions, Bliss, Loewe, QT interval, Clinical trial simulations

## Abstract

Computational modelling is a cornerstone of Comprehensive In Vitro Proarrhythmia Assay and is re-increasingly being used in drug development. Electrophysiological effects of drug-drug interactions can be predicted in silico, e.g. with the use of in vitro cardiac ion channel data, PK profiles and human ventricular cardiomyocyte models. There are, however, several approaches with different assumptions used to assess the combined effect of multiple drugs, and there is no agreed standard interaction model. The aim of this study was to assess whether the choice of the drug-drug interaction (DDI) model (Bliss independence, Loewe additivity, or simple sum) influences the results of QT interval simulation trial. The Simcyp Simulator version 12.1 (Simcyp Ltd. [part of Certara], Sheffield, UK) and Cardiac Safety Simulator 2.0 (Simcyp Ltd. [part of Certara], Sheffield, UK) were used to simulate results of 8 virtual trials mimicking clinical studies and generate individual QTc data. The combined effect of inhibitory actions of drugs which were given simultaneously was calculated with use of three different interaction models. The PD effect of DDI was assessed and the differences between mean observed and mean predicted ΔQTcB values for terfenadine interactions were not statistically significant in all but one cases. Differences between the three DDI models are not statistically significant, implying that the choice of the DDI model, in the case of lack of synergy or antagonism, is irrelevant to the average predicted effect at the clinical level. However, in some cases, it can influence the verdict on combinatorial therapy safety for individual patients.

## Introduction

Polypharmacy is a fact of life in the clinic. Medicine in general and pharmacotherapy, in particular, take advantages of combination therapies in many diseases, e.g. hypertension, diabetes, asthma or cancer. When intended, drug interactions resulting from drug combinations maximize the efficacy of a therapy and minimize adverse effects. On the other hand, however, in the majority of cases, simultaneous use of several active compounds complicates the pharmacotherapy and may lead to increased risk of health- or life-threatening drug-drug interactions (DDI). The mechanism of interaction can be specific for the pharmacokinetics (PK) of the combined drugs as well as their pharmacodynamic (PD) activity or combine both areas—PK/PD. PK interaction can occur at any level of the absorption, distribution, metabolism, and excretion (ADME). The detailed processes vary for a different element of drugs pharmacokinetics [26]. There are plethora of potential interaction mechanisms at the level of drug action. Pharmacodynamic interactions occur when a precipitant drug alters the clinical effects of the object drug at its site of action [4]. This alteration is caused by modification of the environment which finally results in increasing or decreasing the expected clinical effects. One of the potential harmful effects of DDI is the QT interval prolongation and torsade de pointes (TdP) arrhythmia.

DDIs are studied in vitro or in vivo during clinical trials. However, many of potential adverse effects being a consequence of DDI are not revealed until the drug is marketed because of, among other issues, infeasibility of experimental testing of all possible drug combinations. Computational approaches offer an advantage in DDIs evaluation as it is possible to explore wide space of drug combinations using various types of drug data (e.g. chemical structure, physico-chemical properties, information about targets) [3, 12, 24, 27, 33, 34, 36, 37].

In silico modelling is a cornerstone of CiPA (Comprehensive in vitro Proarrhythmia Assay) initiative and currently witnessed proarhythmia assessment paradigm shift. To incorporate DDIs into proarrhythmia modelling, it is necessary to assess the combined effect of multiple drugs on channel or electrocardiogram (ECG) level. There are several approaches used to assess the combined effect of multiple drugs [8]. Proposed DDI models differ in their assumptions, and there is a lack of consensus on the standard interaction model [31]. The Loewe additivity [23] and Bliss independence [2] models are widely used and applied to various problems [11]. The main assumption of the Loewe model is that all the interacting compounds in a combination act on the same target or binding site through the same mechanism while Bliss model assumes exactly the opposite, i.e. drugs in a combination act independently, yet they can perturb their individual responses. Both the models assume the lack of synergistic and antagonistic interaction between drugs. In such situation, there is also the possibility to consider the effect of interaction as a simple sum of the effects of single compounds up to some physiologically feasible maximum level.

The aim of this study was to assess whether the choice of the DDI model influences the results of QT interval simulation trial and which of the applied interaction models generates the results closest to the observed values. Terfenadine was chosen as an exemplary case as there are published results of several clinical studies where different QT prolonging drugs were administered concomitantly with terfenadine.

## Methods

The Simcyp Simulator version 14.1 (Simcyp Ltd. [part of Certara], Sheffield, UK) and Cardiac Safety Simulator 2.0 [10] were used to simulate results of eight virtual trials mimicking clinical studies. It is worth mentioning that these eight trials cover all information on that topic in the publicly available scientific sources [35]. Simcyp platform enabled to estimate drug exposure resulting from DDIs between terfenadine and co-administrated metabolic inhibitors (clarithromycin, erythromycin, fluconazole, fluoxetine, itraconazole, ketoconazole, and paroxetine). The simulations of the pharmacokinetics of terfenadine alone and in the presence of metabolic inhibitor were performed using full physiologically based pharmacokinetic (PBPK) model to allow to simulate the heart tissue concentrations. Pharmacodynamic effects of DDIs in the population of patients were simulated with the use of Cardiac Safety Simulator.

Both PK and PD components of DDI simulation were designed to mimic all settings of the above mentioned clinical trials in terms of demographic, physiological and genetic characteristics of the observed populations. Each study was simulated 10 times to account for the inter-study variability as described previously [35].

Three different interaction models were applied to calculate the combined effect of inhibitory actions of drugs which were given simultaneously, i.e. (1) simple arithmetic sum with a maximal inhibition limit of 1 (all channels blocked in 100 %), (2) Bliss independence, and (3) Loewe additivity to assess which of the applied interaction models generates the results closest to the observed values.

1. Simple sum model.

$$ \begin{array}{l}for\ E\left(A,B\right)\le 1:E\left(A,B\right)=E(A)+E(A);\hfill \\ {}for\ E\left(A,B\right)>1:E\left(A,B\right)=1\hfill \end{array} $$

Equation 2. Bliss independence model [2].

$$ \mathrm{E}\left(\mathrm{A},\mathrm{B}\right)=\mathrm{E}\left(\mathrm{A}\right)+\mathrm{E}\left(\mathrm{B}\right)-\mathrm{E}\left(\mathrm{A}\right)*\mathrm{E}\left(\mathrm{B}\right) $$

Equation 3. Loewe additivity model [23].

$$ \mathrm{E}\left(\mathrm{A},\mathrm{B}\right)=\frac{\mathrm{E}\left(\mathrm{A}\right)+\mathrm{E}\left(\mathrm{B}\right)-2\ast \mathrm{E}\left(\mathrm{A}\right)\ast \mathrm{E}\left(\mathrm{B}\right)}{1-\mathrm{E}\left(\mathrm{A}\right)\ast \mathrm{E}\left(\mathrm{B}\right)} $$

**Fig. 1:**
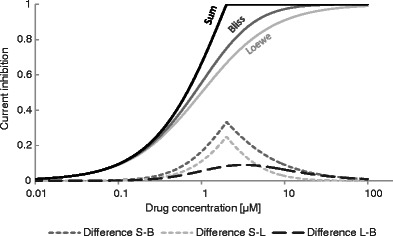
Resultant channel inhibition for 3 DDI models. Equal concentrations and potencies assumed; [DRUG A] = [DRUG B]; IC50 = 1 μM, *n* = 1. *S*—simple sum model; *B*—Bliss model; *L*—Loewe model

Figure 1 presents expected differences in DDI effect predicted by three different models assuming the same concentrations and equal potencies of interacting drugs (*A*, *B*).

For all tested interaction models, the maximum current inhibition was set to 1 (100 %).

The measure of distance (D) was calculated as follows:

$$ \mathrm{D}=\sqrt{{\left(\varDelta {\mathrm{T}}_{\mathrm{pred}}-\varDelta {\mathrm{T}}_{obs}\right)}^2+{\left(\varDelta \mathrm{T}+{\mathrm{I}}_{\mathrm{pred}}-\varDelta \mathrm{T}+{\mathrm{I}}_{obs}\right)}^2+{\left(\left(\varDelta \mathrm{T}+{\mathrm{I}}_{\mathrm{pred}}-\varDelta {\mathrm{T}}_{\mathrm{pred}}\right)-\left(\varDelta \mathrm{T}+{\mathrm{I}}_{obs}-\varDelta {\mathrm{T}}_{obs}\right)\right)}^2} $$

where:

Δ: QT interval change
T: terfenadine
I: inhibitor
pred: predicted value
obs: observed value

It was possible to compare the average values only as the observations for individual patients were not reported in the literature. Predictions for individual patients were analysed separately to determine the relevance of the model choice for the drug cardiac safety assessment. Safety threshold for which assumed DDI model gives results which are of relevance to conclude on potential health-threatening was defined as 5 ms of the heart rate corrected QT prolongation. A mean QTc interval prolongation of 5 ms with the upper limit of 95 % confidence interval above 10 ms is defined as threshold pharmacologic effect of a drug on cardiac repolarization and the threshold level of regulatory concern (ICH [20]). For the sake of this analysis, the case was defined as a situation where using different interaction model would change the decision about meeting the above-defined threshold. The 5 ms threshold can be recognized as very strict, especially considering the expected population variability; however, this was used to mimic the ICH E14 suggested value.

## Results

The simulated and the reported clinical values of area under the curves (AUCs) and maximum plasma concentrations (C_max_) are given in Table 1.

**Table 1:** Comparison of the observed and simulated pharmacokinetic results of the clinical trials

Studied inhibitor (Reference)		C_max_ [ng/ml]		AUC^a^ [ng × h/ml]	
		Observed	Predicted	Observed	Predicted
Fluoxetine [1]	T	2	2.3	24.6	15.3
	T + I	1.4	2.3	14.2	15.3
Erythromycine [15]	T	<5	2.71	NA	NA
	T + I	20.3^b^	9.74	NA	NA
Fluconazole [16]	T	<5	2.48	NA	NA
	T + I	<5	4.11	NA	NA
Itraconazole [17]	T	7.63^c^	4.9	NA	NA
	T + I	14.97^c^	14.6	NA	NA
Ketoconazole [18]	T	7^d^	2.23	NA	NA
	T + I	49.3^e^	19.67	NA	NA
Clarithromycin [19]	T	<5	9^f^	NA	NA
	T + I	2.39	7.03	NA	NA
Erythromycine [19]	T	<5	2.47	NA	NA
	T + I	7.6^g^	8.98	NA	NA
Paroxetine [25]	T	3.68	2.31	30.8	20.5
	T + I	3.64	2.48	30.0	27.5

*T* terfenadine only, *T + I* terfenadine + inhibitor, *NA* not available

^a^Time as in the clinical study

^b^For 3 out of 9 subjects; remaining subjects <5 ng/ml

^c^For 3 out of 6 subjects; remaining subjects <5 ng/ml

^d^For 1 out of 6 subjects; remaining subjects <5 ng/ml

^e^Estimated from the graph for 5 out of 6 subjects; remaining subject <5 ng/ml

^f^For 4 out of 6 subjects

^g^For 3 out of 6 subjects

Table 2 presents results of simulation QT studies and compares them with the reported clinical values. Distance is the measure indicating goodness of fit of the predicted ΔQTc values as compared against the observed one.

**Table 2:** Observed vs. predicted average QT intervals

		QT interval [ms]			
		Observed	Predicted sum^a^	Predicted Bliss^a^	Predicted Loewe^a^
Fluoxetine [1]	BL	372.4	396.0		
	T	374.9	398.5		
	I	–	400.9		
	T + I	379	401.6	400.8	400.2
		Distance	**1.49**	2.59	3.34
Erythromycin [15]	BL		392.8		
	T^2^	8	398.5		
	I^b^	21	403.7		
	T + I^b^	39	418.2	413.6	411.4
		Distance	**18.38**	22.75	25.13
Fluconazole [16]	BL	398.5	395.2		
	T	398.4	401.3		
	I		414.3		
	T + I	411	422.6	418.6	416.8
		Distance	31.28	26.02	**23.65**
Itraconazole^c^ [17]	BL	376	430.5		
	T	390	438		
	I	No effect	486.9		
	T + I	417	452.2	448.9	447.3
		Distance	**24.05**	28.50	30.68
Ketoconazole [18]	BL	408	394.5		
	T	416	395		
	I	–	415.4		
	T + I	490	439	427.9	423.6
		Distance	**48.61**	64.03	70.14
Clarithromycin [19]	BL	409	395.5		
	T	410	401.4		
	I	407	393.9		
	T + I	430	404.4	403.8	402.7
		Distance	**21.43**	22.96	23.79
Erythromycin [19]	BL	394	397.5		
	T	408	403.7		
	I	409	405.8		
	T + I	428	420.1	415.6	413.5
		Distance	**14.27**	19.46	22.17
Paroxetine [25]	BL	381	392.0		
	T	387	394.5		
	I	–	393.5		
	T + I	386	396.6	393.6	396.8
		Distance	**4.69**	4.86	4.78

In bold - the best fitted model (the lowest distance value)

*BL* baseline, *T* terfenadine, *I* inhibitor

^a^DDI effect on ion channels calculation method

^b^Only QT change reported in the study

^c^Maximal QT change

**Fig. 2:**
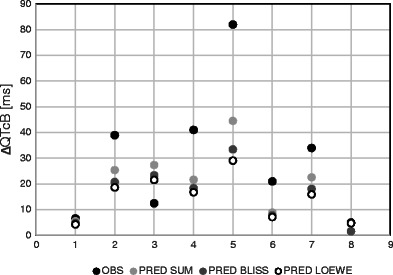
Baseline subtracted changes of QTcB interval—predicted vs. observed

Predicted with the use of three different interaction models and observed average changes of QTcB interval lengths (baseline subtracted) resulting from concomitant administration of terfenadine and an inhibitor are presented on Fig. 2.

Percentage of cases meeting the criterion of reaching the safety threshold (5 ms increase for QTcB) for all analysed models are presented in Table 3.

**Table 3:** Percentage of cases where DDI model choice changes conclusion on QT prolongation safety

DDI study	Fluoxetine [1]	Erythromycin [15]	Fluconazole [16]	Itraconazole [17]	Ketoconazole [18]	Clarithromycin [19]	Erythromycin [19]	Paroxetine [25]
% of cases	0.1	2.8	3.8	2.3	5.6	0.2	3.3	0

## Discussion and Conclusions

The early assessment of cardiac safety liabilities is essential to advance novel drug candidates confidently. Apart from being early, the screening system should also be efficient and cost-effective. Detection of the drug-induced electrophysiological toxic effects is, therefore, crucial. The new paradigm of cardiac safety testing using in vitro human ion channel assays, human-based in silico reconstructions and human stem cell-derived cardiomyocytes has been recently discussed [9]. One of the characteristics of all safety testing systems is their focus on the single chemical entity. Although, as it was described before in the clinical settings, the situation where a single drug is given is relatively rare and polypharmacy is very common. Therefore, system for the safety assessment of the drug combinations would be needed. From the above-listed systems and methods human-based in silico reconstructions of cardiac electrophysiology look best suited for such need. If properly validated, computer simulations with the use of biophysically detailed models can offer precise information about the clinical consequences of drug combinations without running clinical trials, especially when combined with the already commonly used PBPK models used for the exposure assessment.

In the current study, the pseudoECG signals were simulated for terfenadine alone and in the presence of seven different compounds which are inhibitors of terfenadine metabolism as well as known QT prolonging drugs. The pharmacokinetic component of the DDI was modelled and simulated with the use of Simcyp Simulator. The predicted AUC and C_max_ values for terfenadine alone and with the concomitantly given inhibitors were close to the clinically observed values as presented in the recent publication [35]. Predicted terfenadine and inhibitor exposure profiles were utilized to calculate ion channels inhibition and current changes (I_Kr_, I_Ks_, I_Na_, I_Ca_) resulting from drug combination. The latter was done assuming three models of DDI effect. Current specific, maximal ion channel conductances expressed in the ten Tusscher human ventricular cardiomyocyte model were modified by the calculated total inhibition of ion currents. The inhibition was drug concentration dependent, under the assumptions that the in vitro measured activity equals to in vivo situation and that the maximum inhibition cannot exceed 100 %. The ΔQTcB was calculated on the basis of simulated pseudoECG signals for baseline (no drug), terfenadine alone, and terfenadine plus inhibitor scenarios.

The prediction of drug-drug interaction at the PD level was assessed with the use of statistical methods. The differences between mean observed and mean predicted ΔQTcB values for terfenadine interactions were not statistically significant (Welch *t* test) for most of the studies (except for ketoconazole study). Some discrepancies between predictions and clinical observations may arise from the in vitro data variability, small populations of individuals included to the clinical trials, or ΔQTcB calculation method (different definition of baseline).

The hERG channel is the main site of PD interaction between terfenadine and perpetrating drugs used in this study. The Loewe and Bliss DDI model assume the same or different binding sites within channel pore for simultaneously acting channel blockers. Since erythromycin [5, 7] and possibly clarithromycin [6] bind to the external domain of hERG channel protein, while terfenadine [21], fluoxetine [14, 28], fluconazole [13], ketoconazole [29, 30, 32], paroxetine [22] and presumably itraconazole bind to internal domain of hERG channel pore, the Bliss and Loewe model, respectively, should perform better for those groups of drugs. This is not the case according to the results obtained it this study. As indicated in Table 2, in almost all cases, simple sum method yield the closest results to those observed in clinical trials. However, differences between the three DDI models are not statistically significant, implying that the choice of the DDI model, in case of lack of synergy or antagonism, is irrelevant to the average predicted effect at the clinical level.

In the thorough QT (TQT) studies, threshold of regulatory concern for QT prolongation is set as 5 ms (ICH [20]). The percentage of individual patient records, where the difference between the models predictions was at least 5 ms ranged from 0 to 78 % depending on the perpetrating drug. However, only cases where the choice of the model could be of relevance for the conclusions drawn, i.e. where the models predictions differ and put the QTc change on the opposite sites of the safety threshold, can be interpreted as important difference. As shown in Table 3, the percentage of such cases is low. It can be concluded that the model choice can influence the verdict on combinatorial therapy safety for individual patients, but it has a negligible effect at the level of population.
